# Risk Factors and Mental Health Promotion Strategies in Children During COVID-19

**DOI:** 10.3389/fpubh.2020.580720

**Published:** 2020-10-15

**Authors:** Cristiana Alessia Guido, Ilaria Amedeo, Federica Avenoso, Jacopo Bruni, Anna Maria Zicari, Lorenzo Loffredo, Alberto Spalice

**Affiliations:** Paediatric Neurology Division, Department of Maternal Sciences, Sapienza University, Rome, Italy

**Keywords:** risk factors, COVID19, mental health, children, strategies

Since 5 March 2020, the Italian government has ordered a nationwide school closure as an emergency measure to prevent diffusion of the infection. The emergency home-schooling plan has been strictly implemented thanks to the hard work of schools and teachers to create online courses. Furthermore, in a first step, public activities were discouraged, and now, they are firmly forbidden. Researchers have established that such measures should have both negative psychological and physical effects on children. Stressors, such as prolonged duration; fears of infection; frustration and boredom; inadequate information; lack of in-person contact with classmates, friends, and teachers; lack of personal space at home; and family financial loss can have even more problematic and lasting effects on children and adolescents (1).

Evidence suggests that when children are out of school (e.g., weekends and summer holidays), they are physically less active, have much longer screen time, have irregular sleep patterns, and have less favorable diets. Such negative effects on mental health are expected to be much worse when children are confined to their homes without outdoor activities and interaction with friends of the same age (3), which are essential for their normal psychological development and well-being. The uncertainty about the personal and global effects of COVID-19 is creating great concern, in addition to the specific psychological effect of quarantine (2). Some children might be separated from their parents due to the infection. Parental separation pushes children into a state of crisis and might increase the risk of psychiatric disorders, as well as a higher risk of developing mood disorders, psychosis, and death by suicide in adulthood (2). The age of the initial separation is known to be relevant to psychological development disrupting the ongoing attachment processes with significant outcomes if this happens in the first year after birth (3). In this rapidly changing situation, children are experiencing substantial changes to their daily routine. At the same time, they are exposed to large amounts of information and high levels of stress and anxiety in the adults around them (2). Sprang et al. (4) reported that children who were isolated or quarantined during the influenza A (H1N1) pandemic in 2009 were more likely to develop acute stress disorder, adjustment disorder, and pain. Conversely, anxiety in children and adolescents can also manifest itself in challenging externalizing behaviors, such as acting or arguing (3). In Sprang's sample, 30% of children who were isolated or quarantined met the clinical criteria for posttraumatic stress disorder (PTSD) (4). Adult concern about the implications of COVID-19 could impair their ability to recognize and respond to children's ideas or distress (4).

It should be stressed that children may respond in a different way to an outbreak depending on their age. In this respect, the National Child Traumatic Stress Network (NCTSN) has suggested some reactions according to age group, assuming that preschoolers might manifest fear of staying alone, bad dreams, speech difficulties, loss of bladder/bowel control, constipation, bed-wetting, change in appetite, increased temper tantrums, whining, or clinging behaviors. School age children (ages 6–12) might be irritable, plaintive, or aggressive; have nightmares or sleep/appetite disturbance; and show physical symptoms (headaches, stomach aches), withdrawal from peers, loss of interest, competition for the attention of parents, and forgetfulness of household chores and new information learned at school.

Lastly, adolescents (ages 13–18) may complain of physical symptoms (headaches, rashes, etc.), sleep/appetite disturbance, agitation, decrease in energy, or apathy, as well as ignore health promotion behaviors, isolate from peers and loved ones, be concerned about stigma and injustices or avoid/cut school. In this particular contingency, children are exposed to unexplained and unpredictable behavior, which can be perceived as a threat, resulting in a state of anxiety (3). Children are well-attuned to the emotional states of adults; indeed, a strong relationship was found between clinically significant levels of PTSD symptoms in parent respondents and their children; nearly 86% of responders had children who also met the clinical cut-off score (4).

Mental health responses to previous emergencies and disasters have included widespread psychological first aid, focusing on psychoeducation about normative reactions and coping strategies.

Promotional videos can be useful to motivate children to have a healthy lifestyle at home by increasing physical activities and having a balanced diet, regular sleep pattern, and good personal hygiene (1). These are expected to help maintain a daily routine and cope with this difficult moment. In the event of home confinement, parents are often the best and closest resource for children to ask for help; children need honest information about changes; when this information is absent, children try to make sense of the situation on their own (1, 2). They are constantly exposed to news related to the epidemic, so having direct conversations with children about these issues could mitigate their reactions, such as anxiety and panic. Close and open communication with children is also the way to identify any physical and psychological problems, comfort them, and resolve them (1). We agree with Liu and colleagues who propose that pediatric health professionals should receive training to facilitate early identification of children's mental health problems by learning to discern their normal and abnormal behaviors and to use rapid screening tools for mental health (3). Additionally, it is important to consider postpandemic surveillance of mental disorders among these children, remembering that the identification of PTSD or other mental health disorders in parents should trigger an investigation of behavioral health disorders in their family members.

## Author Contributions

CG, IA, FA, and JB wrote the manuscript. AZ and LL revised the manuscript. AS revised and write the manuscript. All authors listed have made a substantial, direct and intellectual contribution to the work, and approved it for publication.

## Conflict of Interest

The authors declare that the research was conducted in the absence of any commercial or financial relationships that could be construed as a potential conflict of interest.
